# Probing Denitrifying Anaerobic Methane Oxidation via Antimicrobial Intervention: Implications for Innovative Wastewater Management

**DOI:** 10.1021/acs.est.3c07197

**Published:** 2024-03-29

**Authors:** Martijn Wissink, Martyna Glodowska, Marnix R. van der Kolk, Mike S. M. Jetten, Cornelia U. Welte

**Affiliations:** † Department of Microbiology, Radboud Institute for Biological and Environmental Sciences, Radboud University, Heyendaalseweg 135, 6525AJ Nijmegen, The Netherlands; ‡ Synthetic Organic Chemistry, Institute for Molecules and Materials, Radboud University, Heyendaalseweg 135, 6525AJ Nijmegen, The Netherlands

**Keywords:** inhibitors, antibiotics, heavy metal pollution, greenhouse gases, wastewater treatment

## Abstract

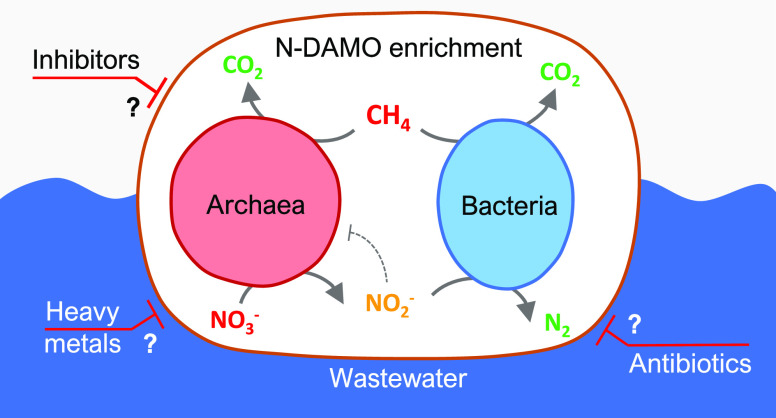

Methane emissions present a significant environmental challenge in both natural and engineered aquatic environments. Denitrifying anaerobic methane oxidation (N-DAMO) has the potential for application in wastewater treatment plants. However, our understanding of the N-DAMO process is primarily based on studies conducted on environmental samples or enrichment cultures using metagenomic approaches. To gain deeper insights into N-DAMO, we used antimicrobial compounds to study the function and physiology of ‘*Candidatus* Methanoperedens nitroreducens’ and ‘*Candidatus* Methylomirabilis oxyfera’ in N-DAMO enrichment cultures. We explored the effects of inhibitors and antibiotics and investigated the potential application of N-DAMO in wastewater contaminated with ammonium and heavy metals. Our results showed that ‘*Ca.* M. nitroreducens’ was susceptible to puromycin and 2-bromoethanesulfonate, while the novel methanogen inhibitor 3-nitrooxypropanol had no effect on N-DAMO. Furthermore, ‘*Ca.* M. oxyfera’ was shown to be susceptible to the particulate methane monooxygenase inhibitor 1,7-octadiyne and a bacteria-suppressing antibiotic cocktail. The N-DAMO activity was not affected by ammonium concentrations below 10 mM. Finally, the N-DAMO community appeared to be remarkably resistant to lead (Pb) but susceptible to nickel (Ni) and cadmium (Cd). This study provides insights into microbial functions in N-DAMO communities, facilitating further investigation of their application in methanogenic, nitrogen-polluted water systems.

## Abstract 2

This study proposes the use of antimicrobial compounds as a tool to unravel microbial physiology and biotechnological potential of denitrifying anaerobic methane oxidation.

## Introduction

Methane, the second most prevalent greenhouse gas globally after CO_2_, poses a serious environmental challenge as a driver of climate warming.^1^ Anaerobic oxidation of methane (AOM) has been found to be an important sink of methane by preventing its emission into the atmosphere.^2^ AOM is mediated by a diverse group of microorganisms, including bacteria from the NC10 phylum (Methylomirabilota) and a polyphyletic group of archaea known as ANME.^3^ These anaerobic methanotrophs are present in natural and engineered water systems, where they couple AOM to various electron acceptors such as nitrate, nitrite, sulfate (in syntrophy with sulfate-reducing bacteria), metal oxides, and humic substances.^4−10^ Despite the increasing number of studies on AOM in recent years, primarily focusing on omics data, detailed physiological studies of anaerobic methanotrophs remain scarce due to the absence of pure cultures.

In freshwater systems, anaerobic methane-oxidizing microorganisms of the genus ‘*Candidatus* Methanoperedens’ and ‘*Candidatus* Methylomirabilis’ are frequently observed.^3^ ‘*Ca.* Methanoperedens nitroreducens’ is an ANME2d archaeon that utilizes a reverse methanogenesis pathway for methane oxidation.^5^ Methane is activated by the enzyme methyl-coenzyme M reductase (MCR) and further converted to CO_2_ while reducing nitrate to nitrite.^11^ On the other hand, ‘*Ca.* Methylomirabilis oxyfera’ employs an intra-aerobic pathway reducing the nitrite that is toxic to ‘*Ca.* M. nitroreducens’ to nitric oxide, followed by dismutation of nitric oxide to nitrogen gas and oxygen.^10,12,13^ Subsequently, this oxygen is directly used by its particulate methane monooxygenase (pMMO) to activate methane.^10,12,13^ The cooperative activities of ‘*Ca.* M. nitroreducens’ and ‘*Ca.* M. oxyfera’ establish an efficient denitrifying anaerobic methane oxidation (N-DAMO) system.^14^

Because of the efficient methane and nitrogen removal, N-DAMO holds potential for application as a bioremediation strategy for methanogenic nitrate-polluted water.^2,14−16^ Consequently, introducing N-DAMO into wastewater treatment plants (WWTPs) has been proposed as a means of methane and nitrogen removal.^2,14,15^ However, in many freshwater ecosystems and WWTPs, concentrations of heavy metals such as lead, nickel, and cadmium are high (Table S1). Yet, the effect of these toxic compounds on N-DAMO efficiency is still unknown.

Therefore, in this study, we used various antimicrobial compounds (antibiotics and inhibitors) to study anaerobic methane oxidation in a complex N-DAMO community. These antimicrobial compounds may be used in the future to ultimately enrich N-DAMO microorganisms by removing flanking nonmethanotrophic community members. At the same time, we evaluated the biotechnological application of N-DAMO by studying the effect of organic solvents, ammonium, and various heavy metals commonly present in wastewaters on the N-DAMO efficiency. To address these knowledge gaps, the effect of different antimicrobial compounds on methane oxidation and nitrate reduction was tested in batch incubations using an N-DAMO enrichment culture. It appeared that ‘*Ca.* M. nitroreducens’ was inhibited by 2-bromoethanesulfonate (2-BES) and puromycin, while ammonium below 10 mM and lead (Pb) had little effect.

## Materials and Methods

### Microbial Strains and Cultivation

#### N-DAMO Bioreactor Operation

This study was performed with granular biomass from an enriched N-DAMO bioreactor culture (see the Supporting Information for a detailed description of bioreactor operations). The culture was fed with a mineral medium containing nitrate as the sole electron acceptor and sparged with methane as the sole electron donor. During this study (∼1 year), the AOM activity varied between 70 and 755 μmol_CH4_ day^–1^ g_DW_^–1^ (Table S2).

#### Methanogenic Archaeal Cultivation Condition

*Methanobacterium formicicum* (MF, DSM 1535) was cultured at 37 °C in a standard CP medium^17^ in 120 mL serum flasks (50 mL medium) and 2 bar H_2_/CO_2_ (80:20, vol/vol). Additional H_2_/CO_2_ (80:20, vol/vol) was added when the pressure dropped below 1 atm.

### Activity Assays with Selected Antimicrobial Compounds

Activity assays were performed in 120 mL serum flasks containing a reactor medium complemented with 20 mM HEPES (pH 7.25) and an initial nitrate concentration of 2–3 mM. 40 mL aliquots of granular biomass were withdrawn anoxically from the bioreactor and washed three times in medium through settling followed by liquid removal. The biomass was resuspended in a final volume of 40 mL. After closing the serum flasks with red butyl rubber stoppers (Terumo, Leuven, Belgium), the headspace was flushed with N_2_/CO_2_ (9:1) for at least 10 min, after which the pressure was set to 1.5 bar. Finally, 20 mL of methane (>99%) was added to the headspace, the pH was adjusted to 7.25 ± 0.1 with anoxic KOH, and bottles were incubated at 30 °C and 200 rpm (New Brunswick Innova 40, Eppendorf, Hamburg, Germany) for approximately 96 h. Different chemical compounds were added prior to incubation in biological triplicates (Table 1). Positive controls consisted of medium with biomass, methane, and nitrate, while negative controls consisted of medium without biomass to account for methane loss during the sampling. To prevent toxic nitrite accumulation, nitrate was added daily to a final concentration between 1 and 5 mM depending on the consumption rate. Methane, nitrate, and nitrite concentrations were measured after 4 h and twice a day throughout the incubation.

**Table 1:** Overview of Antimicrobial Compounds, Their Target of Inhibition, and Applied Concentrations Used in This Study^a^

compound	target of inhibition	reported effect on	applied concentrations
Organic Solvents			
dimethyl sulfoxide (DMSO)			0.1% v/v
ethanol (99.8%)			0.63 mM and 0.01% v/v (=17 mM)
methanol (99.9%)			0.63 mM
Antibiotics			
neomycin sulfate	translation	gram–, archaea	50 μg mL^–1^
bacitracin	cell wall synthesis	gram+, archaea	50 μg mL^–1^
puromycin dihydrochloride	translation	gram+, archaea	10, 50, and 75 μg mL^–1^
streptomycin sulfate	translation	gram–	50 μg mL–1
vancomycin HCl	cell wall synthesis	gram+	50 μg mL–1
ampicillin sodium salt	cell wall synthesis	gram+ and –	50 μg mL–1
kanamycin sulfate	translation	gram+ and −	50 μg mL–1
Inhibitors			
2-bromoethanesulfonic acid sodium salt (2-BES)	MCR	methanogenic archaea	20 mM
3-bromopropanesulfonic acid (3-BPS)	MCR	methanogenic archaea	20 mM
3-nitrooxypropanol	MCR	methanogenic archaea	200 μM
1,7-octadiyne (98%)	pMMO	aerobic methanotrophic bacteria	100 μM
ammonium chloride			1, 10, 20, and 100 mM
Heavy Metals			
Pb(II) chloride (98%)			10, 100, 500, and 1000 μM
Ni(II) chloride hexahydrate			10, 100, 500, and 1000 μM
Cd(II) chloride hemipentahydrate (98%)			10, 100, and 500 μM

a Chemicals were purchased from either Merck Life Sciences N.V., Amsterdam, NL, or VWR International B.V., Amsterdam, NL. 3-NOP was synthesized in-house (see the Supporting Information).

Degradation of 3-nitrooxypropanol (3-NOP) was tested by adding 10 mL of the supernatant of an activity assay with bioreactor biomass containing 200 μM 3-NOP to a 50 mL incubation of*M. formicicum*. As controls, instead of the supernatant, 10 mL of CP medium, 10 mL of CP medium with 30 μM 3-NOP, 10 mL of the supernatant of a regular bioreactor biomass incubation without 3-NOP, and 10 mL of the supernatant of an incubation with 4% paraformaldehyde fixed bioreactor biomass with 200 μM 3-NOP were added. Serum flasks were inoculated with 1 mL of exponentially growing *M. formicicum* preculture in triplicates and incubated at 37 °C for 96 h. Cumulative methane production was measured after 96 h.

### Analytical Methods

Methane consumption was followed by injecting 50 μL of gas-phase samples into a gas chromatograph coupled to a flame ionization detector (Hewlett-Packard 5890, Palo Alto, CA, United States, injector temp 150 °C, oven temp 120 °C) equipped with a Porapak Q100 column. Samples were measured in triplicates, and peaks were automatically integrated using GC ChemStation software (Agilent Technologies, Santa Clara, California, USA). Total methane in liquid and gas was determined using Henry’s law [Henry constant (*H*^cp^) = 1.45 × 10^–5^ mol m^–3^ Pa^–1^]. The methane consumption rate was calculated by linear least-squares regression and normalized on biomass dry weight. The biomass dry weight concentration of the bioreactor was determined by filtering 10 mL granule suspension on predried filters (Whatman glass microfiber filters, grade GF/A, Maidstone, UK) followed by 48 h drying at 80–100 °C. Nitrate and nitrite concentrations were determined using colorimetric test strips (detection limit 10 mg L^–1^, MQuant, Merck, Darmstadt, Germany). The nitrate consumption rate was calculated by dividing the total amount of nitrate consumed by the total incubation time and normalized on biomass dry weight. Inhibition was expressed as a percentage relative to a positive control without antimicrobial compounds and corrected for methane loss through sampling using a negative control with only medium and methane. IC_50_ values were calculated by the linear interpolation of the two concentrations closest to 50% inhibition.

## Results and Discussion

### N-DAMO Community Is Sensitive toward Ethanol and DMSO

**Figure 1:**
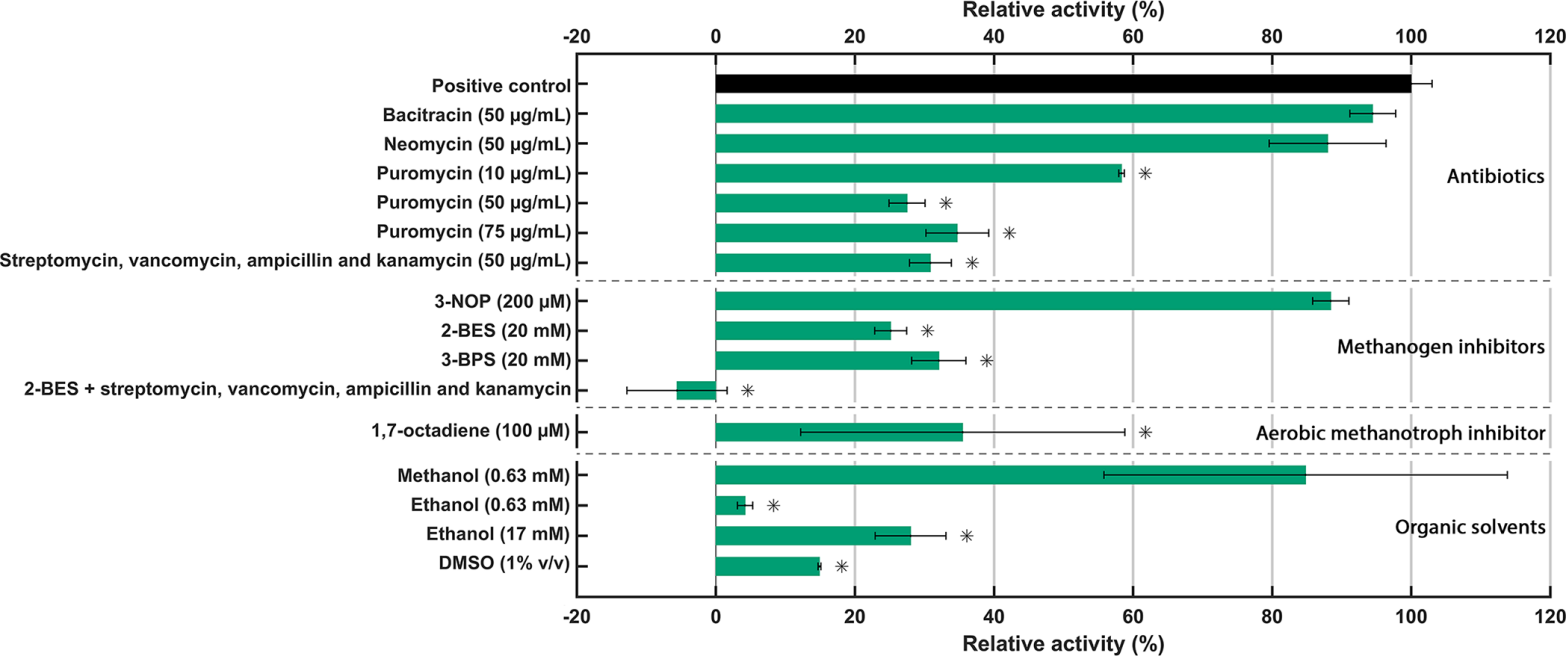
Impact of antimicrobial compounds and organic solvents on the AOM rate. The relative activity of AOM was calculated by dividing the AOM rate (μmol_CH4_ day^–1^ g_dw_^–1^) of incubations supplemented with antimicrobial compounds or solvents by the positive control (with biomass, methane, and nitrate), accounting for the methane loss during sampling in the negative control (with only medium and methane). The methane concentration was measured after 4 h of incubation and measured twice daily over the course of 96 h. Results are obtained from biological triplicates (error bars represent standard deviation). Asterisks indicate a significant difference (*p* < 0.05) compared to the positive control (two-tailed, heteroscedastic *t* test).

In the first part of this study, the complex interactions between anaerobic methanotrophs ‘*Ca.* M. nitroreducens’ (19% abundance in metagenome) and ‘*Ca.* M. oxyfera’ (28% abundance in metagenome) as well as the side community (Figure S3) were investigated using a series of batch experiments with various antibiotics and inhibitors (Table 1). Testing inhibitors and antibiotics can be challenging in the case of moderately or poorly soluble compounds. To administer these compounds, a stock solution in organic solvents such as dimethyl sulfoxide (DMSO), ethanol, or methanol is often prepared. Therefore, we first examined the effect of these organic solvents on the AOM activity. These experiments revealed that the addition of 1% (v/v) DMSO and 0.1% (v/v) ethanol (equivalent to 17 mM) had a significant impact on AOM, resulting in >70% inhibition of AOM (Figure 1). This was a surprising finding considering that DSMO is generally accepted as nontoxic below 10% (v/v) and biological effects of applied concentrations below 2% (v/v) are often unreported as assumed negligible.^18^ For comparison, a study on soil bacterial communities found only minor effects of DMSO in concentrations up to 5% (v/v) with a reduction in the growth rate of up to 20%.^19^ Ethanol, even at low concentrations (0.63 mM), showed an almost complete inhibition of AOM (96 ± 1%) that did not recover within 96 h, which should be sufficient time for the ethanol to be consumed by the side community. Incubations with aerobic methanotrophs have reported similar inhibitory effects after addition of 0.5 mM ethanol, although the exact mechanism remains unclear.^20^ In contrast, methanol at a concentration of 0.63 mM did not exert a significant influence on the AOM rate (Figure 1). The nitrate reduction rate was also significantly decreased by DMSO (52 ± 1% inhibition), while in the case of methanol and ethanol, there was no significant decrease. Nitrate reduction under these conditions can probably be attributed to other nonmethanotrophic community members oxidizing these alcohols (Table S2). All in all, these findings highlight the specific sensitivity of the AOM community to DMSO and ethanol, for which the mechanisms remain to be investigated. For this reason, ethanol and DMSO were avoided as solvents in antibiotic assays in this study and only water-soluble compounds were tested.

### Identification of Effective Antibiotics against Methane-Oxidizing Microorganisms

To assess the contribution of ‘*Ca.* M. nitroreducens’ to methane oxidation and nitrate reduction, antibiotics known to be effective against archaea such as neomycin, bacitracin, and puromycin were tested. We chose antibiotics known to affect methanogens, because of the close phylogenetic relationship and similarities in metabolism between ANME archaea and methanogens.^21^ Neomycin and bacitracin in standard concentrations of 50 μg mL^–1^ did not show a significant effect on methane oxidation and nitrate reduction compared to the positive control (Figure 1 and Table S2). This is in contrast to the methanogenic archaeon *Methanosarcina mazei*, which has been shown to be susceptible to 20 μg mL^–1^ neomycin,^22^ and *Methanobrevibacter smithii*, which is inhibited by 4 μg mL^–1^ bacitracin.^23^ These findings suggest that the N-DAMO community in this study is resistant to neomycin and bacitracin at the concentrations tested.

Puromycin, another antibiotic that is known to affect methanogenic archaea, however, showed significant inhibition at a concentration of 10 μg mL^–1^ with an AOM rate of 42 ± 0.3% (mean ± standard deviation) lower compared to the positive control (Figure 1). Higher concentrations of puromycin (50 and 75 μg mL^–1^) led to further inhibition to 73 ± 3 and 65 ± 4%, respectively. Together, this indicates that the maximum inhibitory effect of puromycin on AOM is ∼70%, which is already reached at a concentration of 50 μg mL^–1^. Since puromycin is effective against archaea and Gram-positive bacteria,^24^ this indicates that the remaining AOM activity can likely be attributed to Gram-negative ‘*Ca.* M. oxyfera’. Nitrate reduction was inhibited to a lesser extent (up to 42 ± 12%, Table S2), which might be facilitated by ‘*Ca.* M. oxyfera’ using storage molecules such as glycogen or by other nonmethanotrophic community members consuming dead biomass. We estimated the concentration of puromycin required to inhibit 50% of the AOM activity (IC_50_) to be <10 μg mL^–1^, taking into account that the maximum effect of puromycin does not completely abolish AOM (Table 2). Ultimately, puromycin sensitivity seems comparable to that of the methanogen *M. mazei*, which is completely inhibited at 5 μg mL^–1^.^22^

**Table 2:** IC_50_ Values for AOM-Affecting Compounds Puromycin, Ammonium, and Heavy Metals

	IC_50_
puromycin	<10 μg mL^–1^
ammonium	52 mM
lead	>1 mM
nickel	0.23 mM
cadmium	<10 μM

To explore the microbial interactions between ‘*Ca.* M. nitroreducens’ and the flanking bacterial community, a bacteria-suppressing antibiotic cocktail containing streptomycin, vancomycin, ampicillin, and kanamycin was used. This cocktail targets cell wall synthesis and translation in both Gram-positive and Gram-negative bacteria potentially inhibiting most of the bacteria in the community.^25^ Batch incubations conducted with this bacteria-suppressing antibiotic cocktail demonstrated a decrease in AOM and nitrate reduction rates of 69 ± 3 and 42 ± 6%, respectively (Figure 1 and Table S2). Since the AOM rate was not completely abolished, this indicates that ‘*Ca.* M. nitroreducens’ is resilient against this bacteria-suppressing antibiotic cocktail and is responsible for the remaining AOM activity. Interestingly, nitrite did not accumulate, which hints toward the use of a possible nitrite detoxification system such as dissimilatory nitrite reduction to ammonium by ‘*Ca.* M. nitroreducens’.^26^ Therefore, the use of this bacteria-suppressing antibiotic cocktail could be an effective strategy to further enrich and isolate ‘*Ca.* Methanoperedenaceae’ in the future.

### Methanogen Inhibitors 2-BES and 3-BPS Seem to Inhibit ‘*Ca.* M. nitroreducens’, while 3-NOP Is Modified or Degraded

A commonly employed strategy to inhibit methanogens is the addition of MCR inhibitors to the growth medium, of which 2-BES is the most frequently used compound. In this study, we aimed to assess the effectiveness of methanogen inhibitors on MCR-containing ‘*Ca.* M. nitroreducens’. Activity assays revealed that 2-BES and its structural analogue 3-bromopropanesulfonate (3-BPS) at 20 mM concentrations similarly inhibited the AOM rate by 75 ± 2 and 68 ± 4%, respectively (Figure 1). Similar to the antibiotic conditions, the nitrate reduction rate was affected less with 41 ± 3 and 14 ± 0% inhibition, respectively (Table S2). The remaining AOM activity and nitrate reduction can likely be attributed to ‘*Ca.* M. oxyfera’ and the side community. In the literature, conflicting observations regarding the effectiveness of 2-BES on ANMEs have been reported. A recent study of ‘*Ca.* M. nitroreducens’ in bioelectrochemical systems revealed a similar inhibition by 20 mM 2-BES as described in this study.^27^ Furthermore, methane oxidation in a culture containing ANME1 and ANME2 was inhibited by 1 mM 2-BES,^28^ while others report that concentrations as high as 50 mM did not show measurable effects on AOM rates, including cultures where ‘*Ca.* M. nitroreducens’ was the dominant species.^5,29,30^ This indicates that the susceptibility of ANME to 2-BES highly depends on the microbial community and/or strain.

A third MCR inhibitor tested was 3-NOP, which has recently emerged as a compound capable of inhibiting MCR at concentrations much lower than 2-BES and 3-BPS.^31^ Notably, 3-NOP has been formulated by Royal DSM under the name Bovaer as a feed additive for ruminants, demonstrating a 30% reduction in biogenic methane emissions by ruminants.^32^ Interestingly, our findings indicate that 3-NOP does not significantly affect AOM at concentrations 20 times higher (200 μM) than required for methanogens (10 μM).^31^ These results suggest that while 3-NOP demonstrates promising efficacy in mitigating methane emissions in ruminants, it is not able to inhibit anaerobic methanotrophic archaea at the concentrations tested in the N-DAMO community here. Whether this is an intrinsic feature of ‘*Ca.* M. nitroreducens’ or a resistance inferred by the flanking community remains unclear.

**Figure 2:**
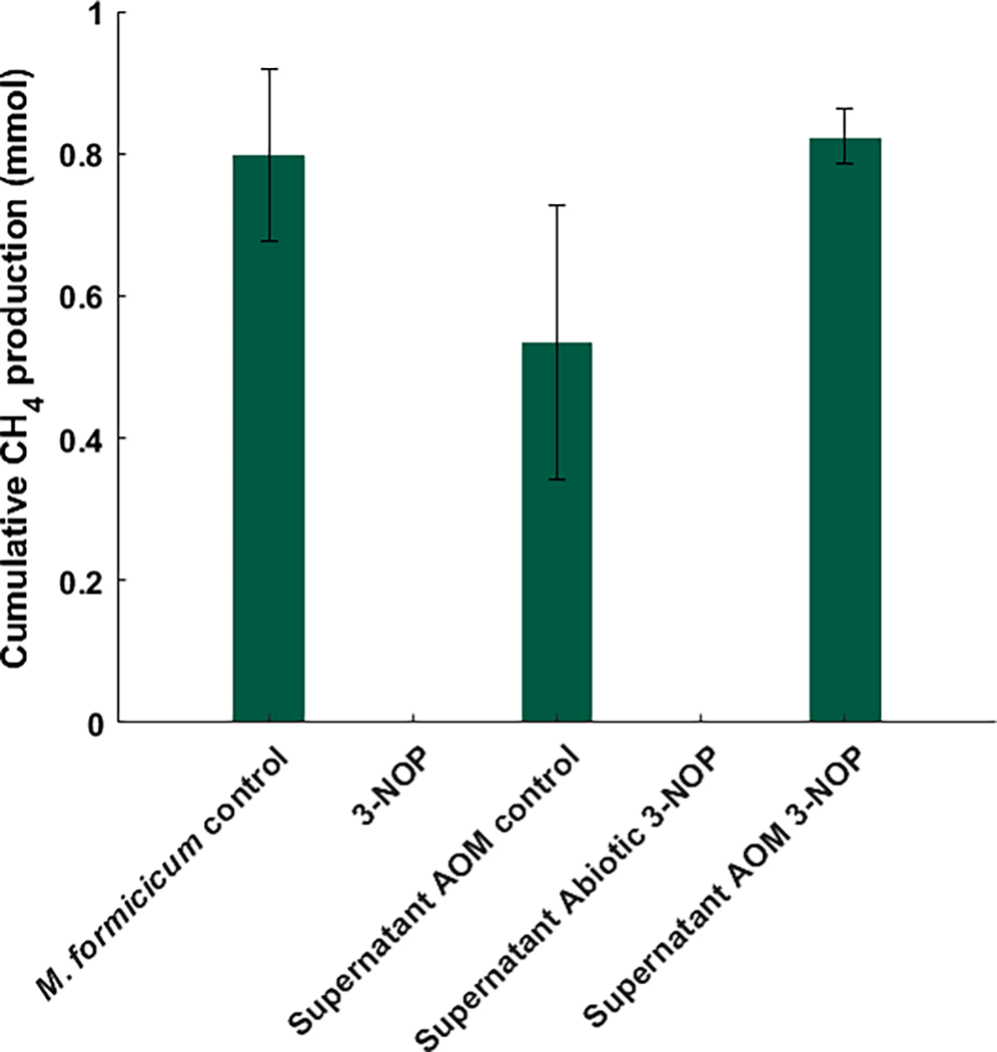
Assessment of degradation or modification of 3-NOP by addition of the N-DAMO community supernatant to *M. formicicum*. 3-NOP was directly added to *M. formicicum* as well as the supernatant obtained from N-DAMO batch cultures which included a control N-DAMO incubation, an abiotic N-DAMO incubation with fixed biomass, and an N-DAMO incubation with 3-NOP. Subsequently, cumulative methane production by *M. formicicum* was monitored for 96 h. Results are averaged from biological triplicates (error bars represent the standard deviation).

A possible resistance mechanism to 3-NOP includes the degradation or modification of 3-NOP. To explore this possibility, we conducted experiments where we added the culture supernatant from a batch incubation with the N-DAMO culture and 200 μM 3-NOP to the medium of the methanogen *M. formicicum*. This way, if 3-NOP was degraded by the N-DAMO community, the supernatant would not affect the growth of *M. formicicum*. In a control with the direct addition of 3-NOP to *M. formicicum*, methane production was completely inhibited (Figure 2). Similarly, the supernatant from an abiotic control with dead N-DAMO biomass and 3-NOP completely inhibited *M. formicicum*, indicating that 3-NOP remained chemically stable throughout the duration of the experiment. Furthermore, the addition of the supernatant from an N-DAMO culture without 3-NOP did not impact methane production by *M. formicicum*. Finally, the addition of the supernatant from an N-DAMO culture with 3-NOP did not affect methane production by *M. formicicum*, implying that the N-DAMO community is capable of degrading or modifying 3-NOP, rendering MCR inhibition ineffective. Further studies would be required to identify degradation or modification mechanisms of 3-NOP.

### 1,7-Octadiyne Seems to Inhibit Methane Oxidation by ‘*Ca.* M. oxyfera’

While MCR inhibitors target ‘*Ca.* M. nitroreducens’, the first step in methane oxidation by ‘*Ca.* M. oxyfera’ relies on the activity of a pMMO. To specifically investigate the role of ‘*Ca.* M. oxyfera’ in the N-DAMO community, we examined the effect of the pMMO inhibitor 1,7-octadiyne (1,7-OD). Batch incubations supplemented with 100 μM 1,7-OD resulted in a significant decrease in the AOM rate of 64 ± 23% accompanied by a decrease in the nitrate reduction rate of 45 ± 7% (Figure 1 and Table S2). This effect was comparable to the incubation with streptomycin, vancomycin, ampicillin, and kanamycin, and also in this case, nitrite did not accumulate. Our findings indicate that 1,7-OD effectively inhibits ‘*Ca.* M. oxyfera’, with results comparable to experiments done with aerobic methanotrophs.^33^

### Syntrophic Relationships Boost the Methane Oxidation Rate

Tested inhibitors exhibited partial effects on methane oxidation, without completely abolishing AOM showing that both ‘*Ca.* M. nitroreducens’ and ‘*Ca.* M. oxyfera’ are important contributors to AOM in the tested cultures. Compounds targeting archaea, such as puromycin, 2-BES, and 3-BPS indeed seemed to inhibit ‘*Ca.* M. nitroreducens’ and reduced the AOM rate by approximately 70%. Similarly, the bacteria-suppressing antibiotic cocktail containing streptomycin, vancomycin, ampicillin, and kanamycin, as well as aerobic methanotroph inhibitor 1,7-OD resulted in a comparable reduction of the AOM rate by approximately 70%. To further investigate archaeal and bacterial contributions to AOM, a combination of 2-BES and the bacteria-suppressing antibiotic cocktail was added to a batch culture to inhibit both methanotrophic bacteria and archaea. The addition of 2-BES-targeting ‘*Ca.* M. nitroreducens’, while the bacteria-suppressing antibiotic cocktail targeted ‘*Ca.* M. oxyfera’, resulted in the complete cessation of methane oxidation (Figure 1). It seems that individually ‘*Ca.* M. nitroreducens’ and ‘*Ca.* M. oxyfera’ can reach an AOM rate of about 30% of the total rate that can be achieved in a syntrophic relationship. These findings demonstrate that unlike sulfate-dependent AOM where ANME form an interdependent syntrophic relationship with sulfate-reducing bacteria,^34^ ‘*Ca.* M. nitroreducens’ and ‘*Ca.* M. oxyfera’ can oxidize methane independently, but rates are greatly enhanced through a syntrophic relationship.

### Response of N-DAMO Community to Various Concentrations of Ammonium and Heavy Metal - Implications for Wastewater Treatment

To explore potential applications of N-DAMO in (waste)water treatment, we investigated the tolerance of the N-DAMO community toward pollutants such as ammonium and heavy metals. Of particular interest were lead (Pb), nickel (Ni), and cadmium (Cd), as these toxic metals are commonly found in wastewaters, often at high concentrations (Table S1).

**Figure 3:**
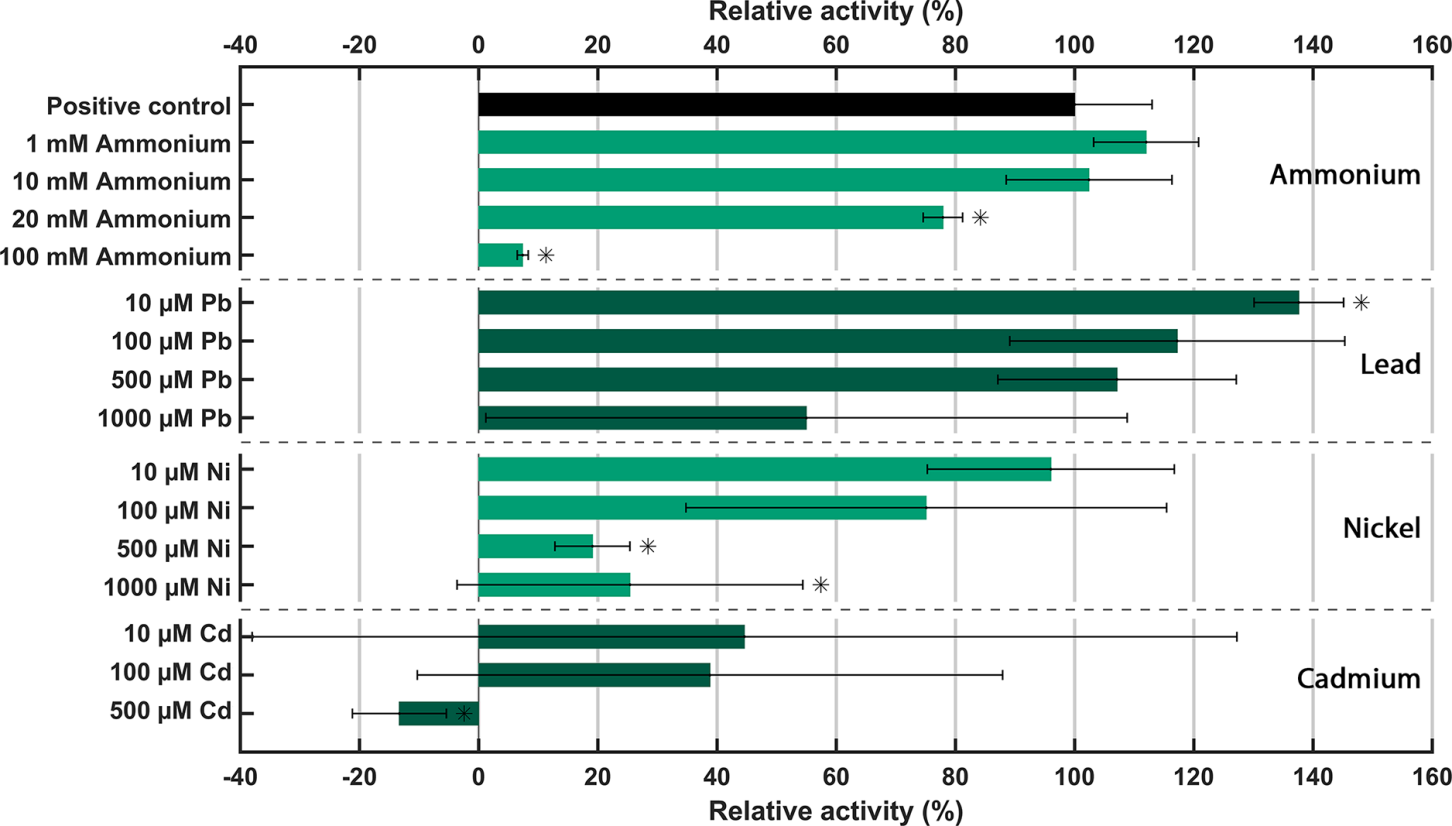
Impact of ammonium, Pb, Ni, and Cd on anaerobic methane oxidation. The relative activity of AOM was calculated by dividing the AOM rate (μmol_CH4_ day^–1^ g_dw_^–1^) of incubation supplemented with ammonium or heavy metals by the positive control (with biomass, methane, and nitrate) accounting for the methane loss during sampling in the negative control (with only medium and methane). The methane concentration was measured after 4 h of incubation and measured twice daily over the course of 96 h. Results are obtained from biological triplicates (error bars represent standard deviation). Asterisks indicate a significant difference (*p* < 0.05) compared to the positive control (two-tailed, heteroscedastic *t* test).

Among the tested heavy metals, the N-DAMO community demonstrated the highest tolerance to Pb (Figure 3). Lead is generally considered toxic to bacteria, as Pb ions can interfere with essential cellular processes and disrupt enzymatic activity. However, some bacteria have developed mechanisms to tolerate or adapt to Pb exposure. Certain bacterial species may possess resistance mechanisms, such as efflux pumps or enzymatic detoxification systems, which allow them to survive in the presence of Pb. The N-DAMO community appeared to be resistant and even benefited from a low concentration of Pb (10 μM), which significantly increased the AOM rate by 38 ± 7%. Concentrations of 100 and 500 μM Pb did not exert a significant impact on the AOM rate, while toxicity effects became apparent at 1000 μM Pb. These observations indicate that the IC_50_ value for Pb exceeds 1000 μM, suggesting a high tolerance to Pb by the N-DAMO community (Table 2). Moreover, the nitrate reduction rates did not decrease at any of the Pb concentrations (Table S2). The significant increase of the AOM rate with 10 μM Pb remains enigmatic but might be caused by introducing a selective advantage for ‘*Ca.* M. nitroreducens’ and ‘*Ca.* M. oxyfera’ over other nonmethanotrophic community members that are likely less resistant to Pb. These data suggest that N-DAMO could be applied in some of the most contaminated environments containing Pb with concentrations up to 336 μM (Table S1).

The effects of Ni and Cd on the N-DAMO community were much more pronounced. In the case of Ni, a concentration of 10 μM did not have a significant effect on the AOM rate. However, at increasing concentrations of 100, 500, and 1000 μM, clear signs of toxicity were observed, resulting in an IC_50_ value of 0.23 mM (Figure 3 and Table 2). Although clear signs of toxicity were observed based on the AOM rate, nitrate reduction was affected much less, which could be attributed to more resistant nonmethanotrophic community members (Table S2). Nickel is used as a cofactor by several microbial enzymes such as urease, hydrogenase, and MCR.^35^ Therefore, it is not surprising that low concentrations of Ni do not have an inhibitory effect on N-DAMO, as this metal is in fact required by some community members, including methanotrophic archaea. However, at high concentrations, Ni becomes toxic to microorganisms as it was shown to be directly involved in the inhibition of enzymes,^35^ which was also the case in our experiment.

In contrast, Cd exhibited pronounced toxicity already at a concentration of 10 μM, completely abolishing methane oxidation at 500 μM. These findings indicate an IC_50_ value for Cd below 10 μM (Figure 3 and Table 2). Similar to Ni, nitrate reduction was affected to a lesser extent (Table S2). Cd is a toxic heavy metal with no known biological function. Besides being an enzyme inhibitor, it can have deleterious effects on the membrane structure and function by binding to ligands such as phosphate and the cysteinyl and histidyl groups of proteins.^36,37^ With concentrations of Ni and Cd being as high as 424 and 8 μM in some wastewaters, respectively (Table S1), the application and efficiency of the N-DAMO process might be hindered.

Similarly, ammonium is a common water contaminant, particularly in agricultural areas. Ammonium was introduced to the batch incubation in concentrations ranging from 1 to 100 mM (Figure 3). The addition of 1 mM ammonium resulted in a marginal increase in the AOM rate, which was not statistically significant. However, a significant decrease in the AOM activity was observed at concentrations of 20 and 100 mM, while the nitrate reduction rate was only affected at 100 mM ammonium (Table S2). It was calculated that the AOM IC_50_ value for ammonium was 52 mM (Table 2). The N-DAMO community appears to be much more sensitive to ammonium compared to methanogenic archaea such as *Methanospirillum hungatei* and *Methanosarcina barkeri* that were shown to resist up to 200 mM NH_4_^+^^38^ and anaerobic digester communities that can tolerate up to ∼150 mM NH_4_^+^.^39^ However, ammonium concentrations in wastewater are typically around 2 mM,^40^ indicating that the presence of ammonium should not be a problem for application of N-DAMO in wastewater treatment.

Using combinations of the antimicrobial compounds, puromycin, a cocktail of bacteria-suppressing antibiotics, 2-BES, 3-BPS, and 1,7-OD allowed us to study and understand the function of N-DAMO community members regarding methane oxidation and nitrate reduction. These antimicrobial compounds can be used as diagnostic tools to study the AOM in environmental samples. Ultimately, the prolonged use of such compounds might lead to the first pure culture isolation of ‘*Ca.* M. nitroreducens’ or ‘*Ca.* M. oxyfera’.

Many studies have suggested the application of N-DAMO in WWTPs. For this, ammonium tolerance is relevant, especially in setups where N-DAMO is combined with anammox. Our study shows that N-DAMO is not affected by low concentrations of ammonium, therefore supporting the possibility of combining N-DAMO with anammox in WWTPs. Moreover, wastewater is often enriched in heavy metals, making it essential to know their effect on N-DAMO. Here, we showed that the N-DAMO community is resistant to Pb up to 1000 μM, but less toward Ni and Cd, although lower concentration of Cd (≤8 μM, Table S1) should be tested to pinpoint Cd thresholds for N-DAMO in wastewater treatment. Ultimately, these results are a step forward toward understanding the application potential of N-DAMO in wastewater treatment.

## Data Availability

The sequencing data associated with this manuscript are deposited in SRA under the BioProject no. PRJNA1011236.

## Supporting Information Available

Additional information on bioreactor cultivation, metagenome sequencing, analysis, and synthesis of 3-NOP; heavy metal concentrations in WWTPs around the world; anaerobic methane oxidation and nitrate reduction rates in batch incubations; ^1^H and ^13^C NMR spectra of synthesized 3-NOP; and microbial community composition (PDF)

## References

[ref1] Dean J. F.; Middelburg J. J.; Röckmann T.; Aerts R.; Blauw L. G.; Egger M.; Jetten M. S. M.; de Jong A. E. E.; Meisel O. H.; Rasigraf O.; Slomp C. P.; in’t Zandt M. H.; Dolman A. J. Methane Feedbacks to the Global Climate System in a Warmer World. Rev. Geophys. 2018, 56 (1), 207–250. 10.1002/2017RG000559.

[ref2] Cai C.; Zhang X.; Wu M.; Liu T.; Lai C. Y.; Frank J.; He B.; Marcellin E.; Guo J.; Hu S.; Yuan Z. Roles and Opportunities for Microbial Anaerobic Oxidation of Methane in Natural and Engineered Systems. Energy Environ. Sci. 2021, 14 (9), 4803–4830. 10.1039/D1EE00708D.

[ref3] Welte C. U.; Rasigraf O.; Vaksmaa A.; Versantvoort W.; Arshad A.; Op den Camp H. J. M.; Jetten M. S. M.; Lüke C.; Reimann J. Nitrate- and Nitrite-Dependent Anaerobic Oxidation of Methane. Environ. Microbiol. Rep. 2016, 8 (6), 941–955. 10.1111/1758-2229.12487. 27753265

[ref4] Raghoebarsing A. A.; Pol A.; Van De Pas-Schoonen K. T.; Smolders A. J. P.; Ettwig K. F.; Rijpstra W. I. C.; Schouten S.; Damsté J. S. S.; Op Den Camp H. J. M.; Jetten M. S. M.; Strous M. A Microbial Consortium Couples Anaerobic Methane Oxidation to Denitrification. Nature 2006, 440 (7086), 918–921. 10.1038/nature04617. 16612380

[ref5] Haroon M. F.; Hu S.; Shi Y.; Imelfort M.; Keller J.; Hugenholtz P.; Yuan Z.; Tyson G. W. Anaerobic Oxidation of Methane Coupled to Nitrate Reduction in a Novel Archaeal Lineage. Nature 2013, 500 (7464), 567–570. 10.1038/nature12375. 23892779

[ref6] Ettwig K. F.; Zhu B.; Speth D.; Keltjens J. T.; Jetten M. S. M.; Kartal B. Archaea Catalyze Iron-Dependent Anaerobic Oxidation of Methane. Proc. Natl. Acad. Sci. U.S.A. 2016, 113 (45), 12792–12796. 10.1073/pnas.1609534113. 27791118 PMC5111651

[ref7] Leu A. O.; Cai C.; McIlroy S. J.; Southam G.; Orphan V. J.; Yuan Z.; Hu S.; Tyson G. W. Anaerobic Methane Oxidation Coupled to Manganese Reduction by Members of the Methanoperedenaceae. ISME J. 2020, 14 (4), 1030–1041. 10.1038/s41396-020-0590-x. 31988473 PMC7082337

[ref8] Cai C.; Leu A. O.; Xie G. J.; Guo J.; Feng Y.; Zhao J. X.; Tyson G. W.; Yuan Z.; Hu S. A Methanotrophic Archaeon Couples Anaerobic Oxidation of Methane to Fe(III) Reduction. ISME J. 2018, 12 (8), 1929–1939. 10.1038/s41396-018-0109-x. 29662147 PMC6052012

[ref9] Valenzuela E. I.; Prieto-Davó A.; López-Lozano N. E.; Hernández-Eligio A.; Vega-Alvarado L.; Juárez K.; García-González A. S.; López M. G.; Cervantes F. J. Anaerobic Methane Oxidation Driven by Microbial Reduction of Natural Organic Matter in a Tropical Wetland. Appl. Environ. Microbiol. 2017, 83 (11), e00645–17 10.1128/AEM.00645-17. 28341676 PMC5440706

[ref10] Ettwig K. F.; Butler M. K.; Le Paslier D.; Pelletier E.; Mangenot S.; Kuypers M. M. M.; Schreiber F.; Dutilh B. E.; Zedelius J.; De Beer D.; Gloerich J.; Wessels H. J. C. T.; Van Alen T.; Luesken F.; Wu M. L.; Van De Pas-Schoonen K. T.; Op Den Camp H. J. M.; Janssen-Megens E. M.; Francoijs K. J.; Stunnenberg H.; Weissenbach J.; Jetten M. S. M.; Strous M. Nitrite-Driven Anaerobic Methane Oxidation by Oxygenic Bacteria. Nature 2010, 464 (7288), 543–548. 10.1038/nature08883. 20336137

[ref11] Berger S.; Cabrera-Orefice A.; Jetten M. S. M.; Brandt U.; Welte C. U. Investigation of Central Energy Metabolism-Related Protein Complexes of ANME-2d Methanotrophic Archaea by Complexome Profiling. Biochim. Biophys. Acta, Bioenerg. 2021, 1862 (1), 148308 10.1016/j.bbabio.2020.148308. 33002447

[ref12] Versantvoort W.; Guerrero-Cruz S.; Speth D. R.; Frank J.; Gambelli L.; Cremers G.; van Alen T.; Jetten M. S. M.; Kartal B.; Op den Camp H. J. M.; Reimann J. Comparative Genomics of Candidatus Methylomirabilis Species and Description of Ca. Methylomirabilis Lanthanidiphila. Front. Microbiol. 2018, 9 (JUL), 1672 10.3389/fmicb.2018.01672. 30140258 PMC6094997

[ref13] Versantvoort W.; Guerrero-Castillo S.; Wessels H. J. C. T.; van Niftrik L.; Jetten M. S. M.; Brandt U.; Reimann J.; Kartal B. Complexome Analysis of the Nitrite-Dependent Methanotroph Methylomirabilis Lanthanidiphila. Biochim. Biophys. Acta, Bioenerg. 2019, 1860 (9), 734–744. 10.1016/j.bbabio.2019.07.011. 31376363

[ref14] van Kessel M. A.; Stultiens K.; Slegers M. F.; Guerrero Cruz S.; Jetten M. S.; Kartal B.; Op den Camp H. J. Current Perspectives on the Application of N-Damo and Anammox in Wastewater Treatment. Curr. Opin. Biotechnol. 2018, 50, 222–227. 10.1016/j.copbio.2018.01.031. 29477927

[ref15] Wang Y.; Wang D.; Yang Q.; Zeng G.; Li X. Wastewater Opportunities for Denitrifying Anaerobic Methane Oxidation. Trends Biotechnol. 2017, 35 (9), 799–802. 10.1016/j.tibtech.2017.02.010. 28283198

[ref16] Legierse A.; Struik Q.; Smith G.; Echeveste Medrano M. J.; Weideveld S.; van Dijk G.; Smolders A. J. P.; Jetten M.; Veraart A. J.; Welte C. U.; Glodowska M. Nitrate-Dependent Anaerobic Methane Oxidation (N-DAMO) as a Bioremediation Strategy for Waters Affected by Agricultural Runoff. FEMS Microbiol. Lett. 2023, 370 (3), fnad041 10.1093/femsle/fnad041. 37170064 PMC10214460

[ref17] Stams A. J. M.; Van Dijk J. B.; Dijkema C.; Plugge C. M. Growth of Syntrophic Propionate-Oxidizing Bacteria with Fumarate in the Absence of Methanogenic Bacteria. Appl. Environ. Microbiol. 1993, 59 (4), 1114–1119. 10.1128/aem.59.4.1114-1119.1993. 16348912 PMC202247

[ref18] Summer K.; Browne J.; Hollanders M.; Benkendorff K. Out of Control: The Need for Standardised Solvent Approaches and Data Reporting in Antibiofilm Assays Incorporating Dimethyl-Sulfoxide (DMSO). Biofilm 2022, 4 (June), 100081 10.1016/j.bioflm.2022.100081. 36060119 PMC9428811

[ref19] Modrzyński J. J.; Christensen J. H.; Brandt K. K. Evaluation of Dimethyl Sulfoxide (DMSO) as a Co-Solvent for Toxicity Testing of Hydrophobic Organic Compounds. Ecotoxicology 2019, 28 (9), 1136–1141. 10.1007/s10646-019-02107-0. 31559559

[ref20] Wieczorek A. S.; Drake H. L.; Kolb S. Organic Acids and Ethanol Inhibit the Oxidation of Methane by Mire Methanotrophs. FEMS Microbiol. Ecol. 2011, 77 (1), 28–39. 10.1111/j.1574-6941.2011.01080.x. 21385187

[ref21] Timmers P. H. A.; Welte C. U.; Koehorst J. J.; Plugge C. M.; Jetten M. S. M.; Stams A. J. M. Reverse Methanogenesis and Respiration in Methanotrophic Archaea. Archaea 2017, 2017, 1–22. 10.1155/2017/1654237. PMC5244752 28154498

[ref22] Mondorf S.; Deppenmeier U.; Welte C. A Novel Inducible Protein Production System and Neomycin Resistance as Selection Marker for Methanosarcina Mazei. Archaea 2012, 2012, 1–8. 10.1155/2012/973743. PMC3407599 22851906

[ref23] Dridi B.; Fardeau M. L.; Ollivier B.; Raoult D.; Drancourt M. The Antimicrobial Resistance Pattern of Cultured Human Methanogens Reflects the Unique Phylogenetic Position of Archaea. J. Antimicrob. Chemother. 2011, 66 (9), 2038–2044. 10.1093/jac/dkr251. 21680581

[ref24] Korrapati M. C. Puromycin. In Encyclopedia of Toxicology; Elsevier, 2014; pp 1141–1142.

[ref25] Imachi H.; Nobu M. K.; Nakahara N.; Morono Y.; Ogawara M.; Takaki Y.; Takano Y.; Uematsu K.; Ikuta T.; Ito M.; Matsui Y.; Miyazaki M.; Murata K.; Saito Y.; Sakai S.; Song C.; Tasumi E.; Yamanaka Y.; Yamaguchi T.; Kamagata Y.; Tamaki H.; Takai K. Isolation of an Archaeon at the Prokaryote-Eukaryote Interface. Nature 2020, 577 (7791), 519–525. 10.1038/s41586-019-1916-6. 31942073 PMC7015854

[ref26] Arshad A.; Speth D. R.; De Graaf R. M.; Op den Camp H. J. M.; Jetten M. S. M.; Welte C. U. A Metagenomics-Based Metabolic Model of Nitrate-Dependent Anaerobic Oxidation of Methane by Methanoperedens-like Archaea. Front. Microbiol. 2015, 6 (DEC), 1–14. 10.3389/fmicb.2015.01423. 26733968 PMC4683180

[ref27] Ouboter H. T.; Mesman R.; Sleutels T.; Postma J.; Wissink M.; Jetten M. S. M.; ter Heijne A.; Berben T.; Welte C. U. Mechanisms of Extracellular Electron Transfer in Anaerobic Methanotrophic Archaea. bioRxiv 2023, bioRxiv:2023.07.24.550278 10.1101/2023.07.24.550278. PMC10874420 38368447

[ref28] Nauhaus K.; Treude T.; Boetius A.; Krüger M. Environmental Regulation of the Anaerobic Oxidation of Methane: A Comparison of ANME-I and ANME-II Communities. Environ. Microbiol. 2005, 7 (1), 98–106. 10.1111/j.1462-2920.2004.00669.x. 15643940

[ref29] Orcutt B.; Samarkin V.; Boetius A.; Joye S. On the Relationship between Methane Production and Oxidation by Anaerobic Methanotrophic Communities from Cold Seeps of the Gulf of Mexico. Environ. Microbiol. 2008, 10 (5), 1108–1117. 10.1111/j.1462-2920.2007.01526.x. 18218032

[ref30] Dekas A. E.; Poretsky R. S.; Orphan V. J. Deep-Sea Archaea Fix and Share Nitrogen in Methane-Consuming Microbial Consortia. Science 2009, 326 (5951), 422–426. 10.1126/science.1178223. 19833965

[ref31] Duin E. C.; Wagner T.; Shima S.; Prakash D.; Cronin B.; Yáñez-Ruiz D. R.; Duval S.; Rümbeli R.; Stemmler R. T.; Thauer R. K.; Kindermann M. Mode of Action Uncovered for the Specific Reduction of Methane Emissions from Ruminants by the Small Molecule 3-Nitrooxypropanol. Proc. Natl. Acad. Sci. U.S.A. 2016, 113 (22), 6172–6177. 10.1073/pnas.1600298113. 27140643 PMC4896709

[ref32] Royal DSM. Taking Action on Climate Change, Together; Royal DSM, 2022.

[ref33] Sakoula D.; Smith G. J.; Frank J.; Mesman R. J.; Kop L. F. M.; Blom P.; Jetten M. S. M.; van Kessel M. A. H. J.; Lücker S. Universal Activity-Based Labeling Method for Ammonia- and Alkane-Oxidizing Bacteria. ISME J. 2022, 16 (4), 958–971. 10.1038/s41396-021-01144-0. 34743174 PMC8941013

[ref34] Scheller S.; Yu H.; Chadwick G. L.; McGlynn S. E.; Orphan V. J. Artificial Electron Acceptors Decouple Archaeal Methane Oxidation from Sulfate Reduction. Science 2016, 351 (6274), 703–707. 10.1126/science.aad7154. 26912857

[ref35] Macomber L.; Hausinger R. P. Mechanisms of Nickel Toxicity in Microorganisms. Metallomics 2011, 3 (11), 1153 10.1039/c1mt00063b. 21799955 PMC4130172

[ref36] Collins Y. E.; Stotzky G. Factors Affecting the Toxicity of Heavy Metals to Microbes. In Metal Ions and Bacteria; Wiley: Toronto, ON, 1989; pp 31–90.

[ref37] Vig K.; Megharaj M.; Sethunathan N.; Naidu R. Bioavailability and Toxicity of Cadmium to Microorganisms and Their Activities in Soil: A Review. Adv. Environ. Res. 2003, 8 (1), 121–135. 10.1016/S1093-0191(02)00135-1.

[ref38] Sprott G. D.; Patel G. B. Ammonia Toxicity in Pure Cultures of Methanogenic Bacteria. Syst. Appl. Microbiol. 1986, 7 (2–3), 358–363. 10.1016/S0723-2020(86)80034-0.

[ref39] Yenigün O.; Demirel B. Ammonia Inhibition in Anaerobic Digestion: A Review. Process Biochem. 2013, 48 (5–6), 901–911. 10.1016/j.procbio.2013.04.012.

[ref40] McCarty P. L. What Is the Best Biological Process for Nitrogen Removal: When and Why?. Environ. Sci. Technol. 2018, 52 (7), 3835–3841. 10.1021/acs.est.7b05832. 29510030

