# Supplementary material for: Probing Denitrifying Anaerobic Methane Oxidation via Antimicrobial Intervention: Implications for Innovative Wastewater Management
Source: Environ Sci Technol. 2024 Mar 29;58(14):6250–7. doi: 10.1021/acs.est.3c07197 (PMC11008094; doi:10.1021/acs.est.3c07197)
Supplement: Supplementary file 1 — es3c07197_si_001.pdf [file es3c07197_si_001.pdf]

Supporting Information to

Probing Denitrifying Anaerobic Methane Oxidation via Antimicrobial Intervention: Implications for Innovative Wastewater Management

Martijn Wissink[1], Martyna Glodowska[1], Marnix R. van der Kolk[2], Mike S.M. Jetten[1], Cornelia U. Welte[1*]

[1] Department of Microbiology, Radboud Institute for Biological and Environmental Sciences, Radboud University, Heyendaalseweg 135, 6525AJ Nijmegen, the Netherlands

[2] Synthetic Organic Chemistry, Institute for Molecules and Materials, Radboud University, Heyendaalseweg 135, 6525AJ Nijmegen, the Netherlands

[*]Address correspondence to Cornelia U. Welte, E-mail address: c.welte@science.ru.nl

Summary: 8 pages, supplementary methods, 2 tables, 3 figures

Supplementary Methods

Bioreactor cultivation

This study was performed with granular biomass from a highly enriched N-DAMO bioreactor culture [1]. The culture was maintained in an anaerobic 15L sequencing fed batch reactor (30°C, pH 7.3±0.1, stirred at 200 rpm) with a working volume between 8 and 11 L. The reactor was continuously sparged with 15 mL min^{-1} CH_4/CO_2 (95:5) and fed with medium (flow rate 2-2.5 L day^{-1}) as described in [2] containing nitrate (15-25 mM) with an adaptation of the KH_2PO_4 concentration to 0.05 g L^{-1}. Once a day the biomass settled for 5 min after which the excess liquid in the reactor was pumped off to a volume of 8 L. During this study (~1 year) the AOM activity varied between 70 to 755 μmol_{CH4} day^{-1} g_{DW}^{-1} (Table S1).

DNA sequencing and community composition analysis

DNA was extracted from bioreactor biomass as described in [3]. One ng of DNA was used for Illumina library construction using the Nextera XT Library Preparation Kit (Illumina, San Diego, California, USA). The libraries were checked for quality and size distribution using the Agilent 2100 Bioanalyzer and the High sensitivity DNA kit (Agilent Technologies, Santa Clara, California, USA). Quantitation of the library was performed by Qubit using the Qubit dsDNA HS Assay Kit (Thermo Fisher Scientific Inc Waltham USA). Libraries were normalized, pooled and paired-end sequenced (2x 300bp) using the Illumina Miseq (Illumina) and the MiSeq Reagent Kit v3 (Illumina) according the manufacturers' protocol. The microbial community compositions was analysed based on rapid small-subunit rRNA profiling and targeted assembly from the Illumina paired-end reads using phyloFlash [4]. Species were annotated using the SILVA database version 138.1 [5].

Synthesis of 3-NOP

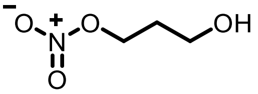

To a solution of 3-bromopropan-1-ol (500 mg, 325 µL, 1.0 Eq, 3.60 mmol) in MeCN (7 mL) was added silver nitrate (911 mg, 1.49 Eq, 5.36 mmol) and the reaction was stirred for 24h at 21 °C. Then another portion of silver nitrate (911 mg, 1.49 Eq, 5.36 mmol) was added and the reaction stirred for another 3 h at 21 °C. The reaction was filtered, evaporated under reduced pressure and purified by column chromatography (Hept : AcOEt 100:0 → 20:80) to yield 3-nitrooxypropanol (355 mg, 2.93 mmol, 81.4 %) as a light yellow oil. **^{1}H-NMR** (500 MHz, $CDCl_3$) δ 4.62 (t, *J* = 6.3 Hz, 2H), 3.79 (td, *J* = 5.9, 4.4 Hz, 2H), 1.99 (p, *J* = 6.2 Hz, 2H), 1.47 (t, *J* = 4.7 Hz, 1H). **^{13}C-NMR** (126 MHz, $CDCl_3$) δ 70.2, 58.8, 29.7.

NMR spectra were recorded at 298 K on a Bruker Avance 500 with cryoprobe (500 MHz) spectrometer in the indicated solvent. Chemical shifts are given in parts per million (ppm) with respect to tetramethylsilane (0.00 ppm) as internal standard for ^{1}H-NMR and $CDCl_3$ (77.16 ppm) as internal standard for ^{13}C-NMR. Purification was performed using a Biotage Isolera One Flash Chromatography system.

The sample was dissolved in $CDCl_3$ from a brand-new bottle that contained no water as determined by a blank ^{1}H-NMR measurement (Fig. S1). Given that both water and the methylene carbons have two protons, the relative wt% of H_2O in the sample could be determined as follows: Integration of the ^{1}H-NMR water signal peak (0.15) and multiplication by the molecular weight (18.02). This was subsequently divided by the product of the integral of the of the 3-nitrooxy propanol peak (2.00) and the molecular weight (121.09) which resulted in a relative wt% of 1.1%.

Supplementary Tables

Table S1. Increased heavy metal concentrations in wastewater treatment plants around the world.

	**Cd** **[mg/L] / *[µM]***	**Pb** **[mg/L] / *[µM***	**Ni** **[mg/L] / *[µM***	**Source**
**WHO drinking water limits**	0.003 / *0.03*	0.01 / *0.05*	0.07 / *1.2*	[6]
**Gdansk, Poland**	0.02 / *0.2*	0.05 / *0.2*	0.770 / *13*	[7]
**Kenya**	0.00009 / *0.0008*	0.01531 / *0.07396*	0.00496 / *0.0845*	[8]
**Marrakeh, Morocco**	0.0757 / *0.673*	1.417 / *6.845*		[9]
**Thessaloniki, Greece**	0.0033 / *0.29*	0.039 / *0.19*	0.78 / *13*	[10]
**Thessaloniki, Greece (in sludge)**	0.001 / *0.009*	0.028 / *0.14*	0.037 / *0.63*	[10]
**Stockholm, Sweden**		0.0006 / *0.003*	0.0071 / *0.12*	[11]
**Beijing, China**	0.84 / *7.5*	49.4 / *239*	24.9 / *424*	[12]
**Sao Paulo, Brazil**	0.00015 / *0.0013*	0.037 / *0.18*	-	[13]
**Cairo, Egypt (treated wastewater)**	0.002 / *0.02*	0.01/ *0.05*	-	[14]
**Peninsular Malaysia**		69.46 / *335.6*	4.8 / *82*	[15]
**Linglong Gold Mining Area, China**	0.194 / *1.73*	0.449 / *2.17*	-	[16]

Table S2. Anaerobic methane oxidation (AOM) and nitrate reduction rates in batch incubations. Rates are normalised by dry weight (DW). The relative activity of AOM was calculated by dividing the AOM rate (μmol_{CH4} day^{-1} g_{dw}^{-1}) of incubation supplemented with antimicrobial compounds by the positive control (with biomass, methane, and nitrate) accounting for the methane loss during sampling in the negative control (with medium and only methane). The relative nitrate reduction activity was calculated similarly, but without negative control. Groups of batch incubations that were performed together are displayed within the horizontal lines. Results are obtained from biological triplicates. P-values are obtained from a two-tailed, heteroscedastic t-test and underlined if p<0.05.

Compound	AOM rate (μmol_{CH4} day^{-1} g_{dw}^{-1})	Relative AOM activity	p-value	NO_3^- reduction rate ($mmol_{NO3-}$ day^{-1} g_{dw}^{-1})	Relative NO_3^- reduction activity	p-value
Positive control (biomass, CH_4, NO_3^-)	299±7	100%±3		0.49±0.02	100%±3	
Puromycin (10 µg mL^{-1})	194±1	58%±0	1.3E-03	0.39±0.02	80%±4	2.71E-03
Streptomycin, vancomycin, ampicillin and kanamycin (50 µg mL^{-1})	125±7	31%±3	8.1E-06	0.28±0.03	58%±6	1.02E-03
Negative control (CH_4)	48±7	0%±3		-	-	
Positive control (biomass, CH_4, NO_3^-)	439±30	100%±7		0.73±0.04	100%±6	
Neomycin (50 µg mL^{-1})	386±37	88%±8	2.4E-01	0.55±0.24	76%±33	3.31E-01
Puromycin (50 µg mL^{-1})	119±11	27%±3	1.0E-03	0.43±0.09	58%±12	1.32E-02
Bacitracin (50 µg mL^{-1})	415±14	94%±3	3.0E-01	0.74±0.02	102%±3	6.77E-01
2-Bromoethanesulfonate (2-BES, 20 mM)	109±10	25%±2	1.2E-03	0.43±0.02	59%±3	1.79E-03
3-Bromopropanesulfonate (3-BPS, 20 mM)	140±17	32%±4	4.7E-04	0.63±0.00	86%±0	5.43E-02
Negative control (CH_4)	-2±8	0%±2		-	-	
Positive control (biomass, CH_4, NO_3^-)	526±30	100%±7		0.77±0.01	100%±1	
Streptomycin, vancomycin, ampicillin, kanamycin (50 µg mL^{-1}) + 2-BES (20mM)	54±32	-6%±7	2.7E-04	0.35±0.03	46%±4	1.40E-03
Negative control (CH_4)	79±5	0%±1		-	-	
Positive control (biomass, CH_4, NO_3^-)	593±45	100%±8		1.04±0.02	100%±2	
3-Nitrooxypropanol (3-NOP, 200 µM)	529±15	88%±3	1.1E-01	0.91±0.02	87%±2	2.00E-03
Puromycin (75 µg mL^{-1})	231±25	35%±4	1.0E-03	0.87±0.08	84%±8	5.85E-02
Ammonium (100 mM)	80±5	7%±1	2.4E-03	0.58±0.08	56%±8	6.62E-03
Negative control (CH_4)	39±3	0%±0		-	-	
Positive control (biomass, CH_4, NO_3^-)	755±45	100%±6		1.40±0.02	100%±1	
Dimethyl sulfoxide (DMSO, 1% v/v)	147±2	15%±0	7.0E-04	0.67±0.01	48%±1	1.37E-05
Ethanol (0.1% v/v = 17 mM)	241±37	28%±5	2.4E-05	1.48±0.00	106%±0	1.07E-02
Negative control (CH_4)	41±29	0%±4		-	-	
Positive control (biomass, CH_4, NO_3^-)	222±27	100%±13		0.54±0.09	100%±16	
1,7-Octadiyne (100 µM)	88±49	36%±23	5.9E-03	0.30±0.04	55%±7	2.64E-02
1 mM Ammonium	247±18	112%±9	2.5E-01	0.56±0.00	103%±0	7.51E-01
10 mM Ammonium	227±29	102%±14	7.4E-01	0.64±0.04	119%±8	1.73E-01
20 mM Ammonium	176±7	78%±3	3.5E-02	0.56±0.00	103%±0	7.51E-01
Negative control (CH_4)	13±8	0%±4		-	-	
Positive control (biomass, CH_4, NO_3^-)	69±12	100%±18		0.47±0.01	100%±2	
500 µM Cd	-6±5	-13%±8	2.8E-03	0.26±0.05	55%±18	1.24E-02
100 µM Cd	29±32	39%±49	1.5E-01	0.36±0.13	77%±35	2.69E-01
10 µM Cd	32±54	45%±83	3.7E-01	0.29±0.20	62%±68	2.52E-01
1000 µM Pb	39±35	55%±54	2.8E-01	0.61±0.00	129%±1	1.00E-03
500 µM Pb	74±13	107%±20	6.7E-01	0.58±0.03	122%±5	1.80E-02
100 µM Pb	80±18	117%±28	4.3E-01	0.50±0.01	106%±2	2.14E-02
10 µM Pb	94±5	138%±7	4.9E-02	0.49±0.00	104%±0	7.87E-02
1000 µM Ni	20±19	25%±29	2.7E-02	0.39±0.15	82%±38	4.29E-01
500 µM Ni	16±4	19%±6	8.8E-03	0.32±0.05	68%±14	2.41E-02
100 µM Ni	52±27	75%±40	4.1E-01	0.50±0.07	107%±14	5.14E-01
10 µM Ni	66±14	96%±21	8.1E-01	0.49±0.07	104%±13	6.82E-01

Negative control (CH_4)	3±3	0%±4		-	-	
Positive control (biomass, CH_4, NO_3^-)	107±19	100%±20		0.17±0.17	100%±99	
Methanol 0.63 mM	92±28	85%±29	5.0E-01	0.25±0.13	148%±50	5.80E-02
Ethanol 0.63 mM	13±1	4%±1	1.4E-02	0.16±0.07	93%±47	9.21E-01
Control (biomass, NO_3^-)	-	-		0.07±0.01	38%±14	3.91E-01
Negative control (CH_4)	9±5	0%±5		-	-	

Supplementary Figures

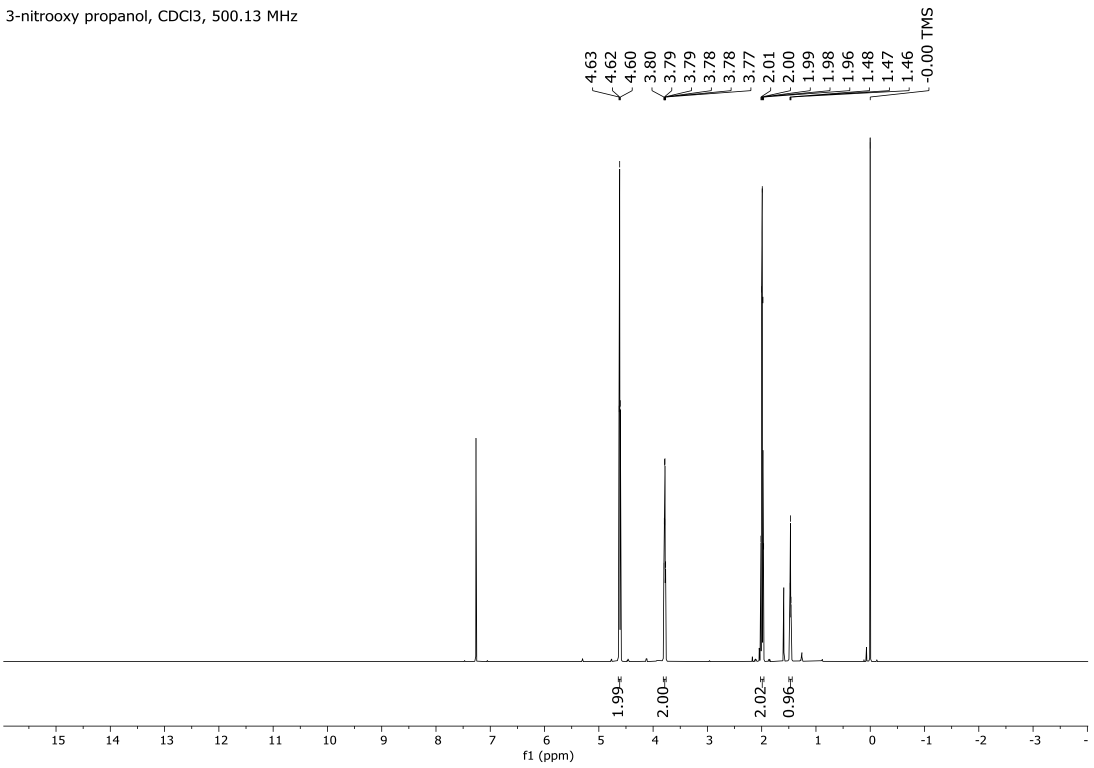

Figure S1. ^{1}H-NMR spectrum of synthesised 3-nitrooxypropanol.

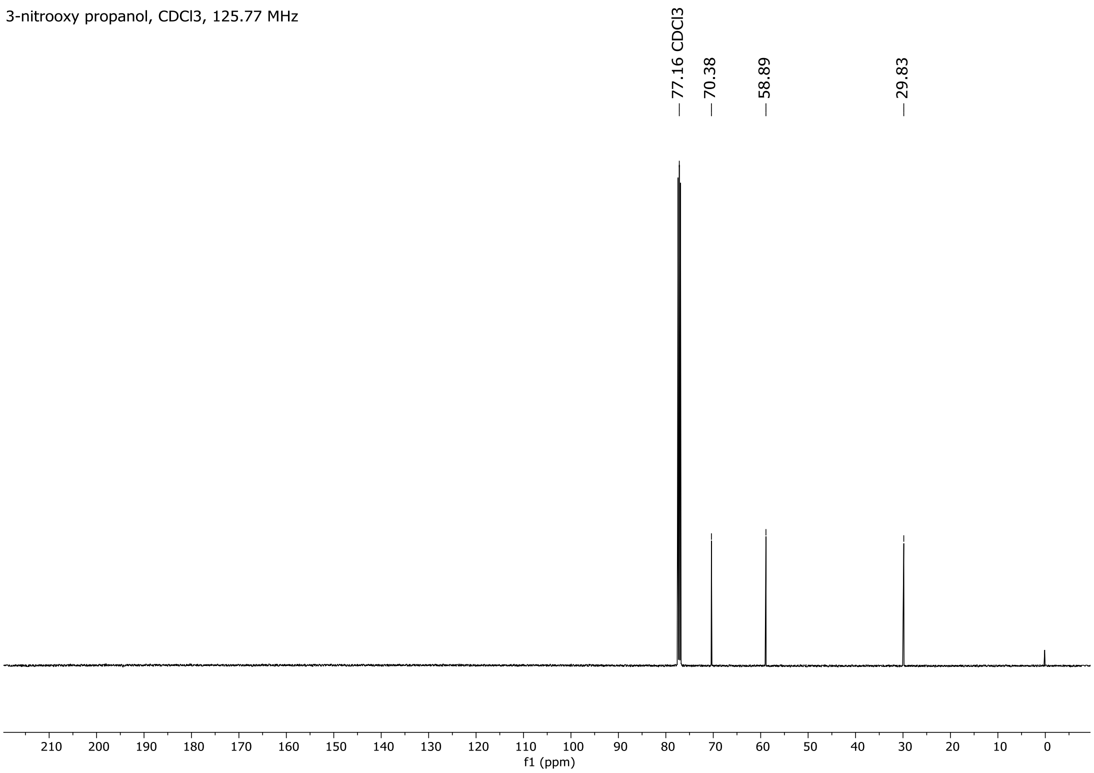

Figure S2. ^{13}C-NMR spectrum of synthesised 3-nitrooxypropanol.

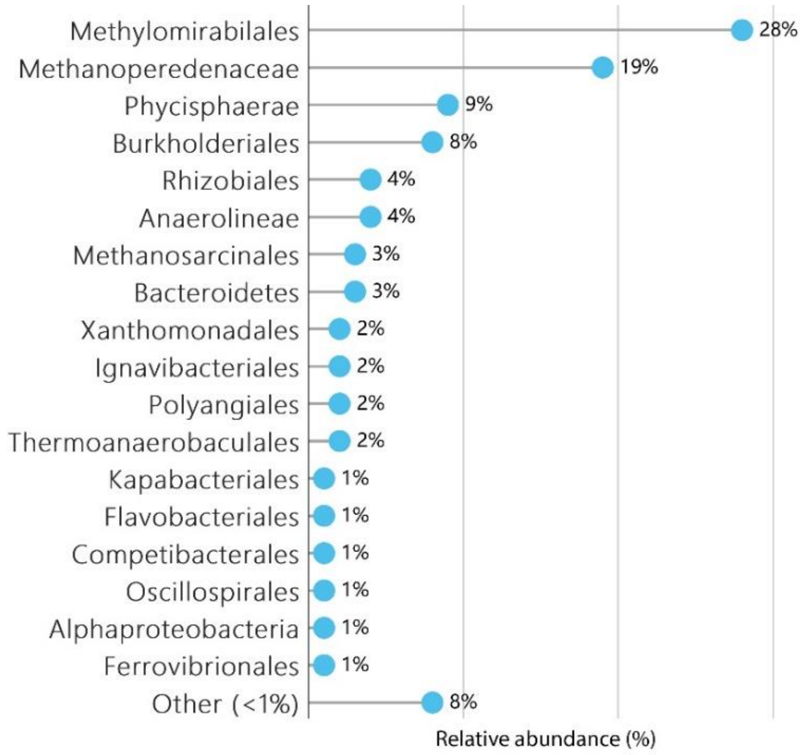

Figure S3. Microbial community composition as determined by Illumina metagenome sequencing and analysed using phyloFlash.

References

1. Berger, S., Cabrera-Orefice, A., Jetten, M. S. M., Brandt, U. & Welte, C. U. Investigation of central energy metabolism-related protein complexes of ANME-2d methanotrophic archaea by complexome profiling. *Biochim Biophys Acta Bioenerg* **1862**, 148308 (2021).
2. Kurth, J. M. *et al.* Anaerobic methanotrophic archaea of the ANME-2d clade feature lipid composition that differs from other ANME archaeaKurth, J. M., Smit, N. T., Berger, S., Schouten, S., Jetten, M. S. M., & Welte, C. U. (2019). Anaerobic methanotrophic archaea of the ANME-. *FEMS Microbiol Ecol* **95**, 1–11 (2019).
3. Ouboter, H. T. *et al.* Methane-Dependent Extracellular Electron Transfer at the Bioanode by the Anaerobic Archaeal Methanotroph "Candidatus Methanoperedens". *Front Microbiol* **13**, 820989 (2022).
4. Gruber-Vodicka, H. R., Seah, B. K. B. & Pruesse, E. phyloFlash: Rapid Small-Subunit rRNA Profiling and Targeted Assembly from Metagenomes. *mSystems* **5**, 10.1128/msystems.00920-20 (2020).
5. Quast, C. *et al.* The SILVA ribosomal RNA gene database project: Improved data processing and web-based tools. *Nucleic Acids Res* **41**, 590–596 (2013).
6. World Health Organization. *WHO Guidelines for Drinking-Water Quality*. vol. 1 (World Health Organization, Geneva, 2004).
7. Chipasa, K. B. Accumulation and fate of selected heavy metals in a biological wastewater treatment system. *Waste Management* **23**, 135–143 (2003).
8. Kinuthia, G. K. *et al.* Levels of heavy metals in wastewater and soil samples from open drainage channels in Nairobi, Kenya: community health implication. *Sci Rep* **10**, 8434 (2020).
9. Chaoua, S., Boussaa, S., El Gharmali, A. & Boumezzough, A. Impact of irrigation with wastewater on accumulation of heavy metals in soil and crops in the region of Marrakech in Morocco. *Journal of the Saudi Society of Agricultural Sciences* **18**, 429–436 (2019).
10. Karvelas, M., Katsoyiannis, A. & Samara, C. Occurrence and fate of heavy metals in the wastewater treatment process. *Chemosphere* **53**, 1201–1210 (2003).
11. Sörme, L. & Lagerkvist, R. Sources of heavy metals in urban wastewater in Stockholm. *Science of the Total Environment* **298**, 131–145 (2002).
12. Wang, X. J. Kriging and heavy metal pollution assessment in wastewater irrigated agricultural soil of Beijing's eastern farming regions. *Journal of Environmental Science and Health, Part A* **33**, 1057–1073 (1998).
13. da Silva Oliveira, A. *et al.* Heavy metals in untreated/treated urban effluent and sludge from a biological wastewater treatment plant. *Environ Sci Pollut Res Int* **14**, 483 (2007).
14. Osman, H. E. M., Abdel-Hamed, E. M. W., Al-Juhani, W. S. M., Al-Maroai, Y. A. O. & El-Morsy, M. H. E.-M. Bioaccumulation and human health risk assessment of heavy metals in food crops irrigated with freshwater and treated wastewater: a case study in Southern Cairo, Egypt. *Environmental Science and Pollution Research* **28**, 50217–50229 (2021).
15. Ashraf, M. A., Maah, M. J. & Yusoff, I. B. Study of Water Quality and Heavy Metals in Soil & Waterof Ex-Mining Area Bestari Jaya, Peninsular Malaysia. *Internation Journal of Basic & Applied Sciences IJBAS-IJENS* **10**, 7–23 (2010).

16. Ning, L., Liyuan, Y., Jirui, D. & Xugui, P. Heavy Metal Pollution in Surface Water of Linglong Gold Mining Area, China. *Procedia Environ Sci* **10**, 914–917 (2011).
